# Federated learning frameworks: quality and interoperability for biomedical research

**DOI:** 10.1093/nargab/lqag010

**Published:** 2026-02-02

**Authors:** María Chavero-Diez, Carles Hernandez-Ferrer, Laia Codó, Josep Ll Gelpí, Salvador Capella-Gutiérrez

**Affiliations:** Barcelona Supercomputing Center (BSC), Plaça d’Eusebi Güell, Barcelona E-08034, Spain; Biochemistry and Molecular Biomedicine Department, University of Barcelona Av. Diagonal 643, Barcelona E-08028, Spain; Barcelona Supercomputing Center (BSC), Plaça d’Eusebi Güell, Barcelona E-08034, Spain; Barcelona Supercomputing Center (BSC), Plaça d’Eusebi Güell, Barcelona E-08034, Spain; Barcelona Supercomputing Center (BSC), Plaça d’Eusebi Güell, Barcelona E-08034, Spain; Biochemistry and Molecular Biomedicine Department, University of Barcelona Av. Diagonal 643, Barcelona E-08028, Spain; Barcelona Supercomputing Center (BSC), Plaça d’Eusebi Güell, Barcelona E-08034, Spain

## Abstract

This review examines the current landscape of federated learning frameworks to evaluate their long-term sustainability, flexibility, and usability in biomedical research, where strict data regulations limit data sharing across institutions. Through a systematic literature analysis, the study assesses these frameworks against findability, accessibility, interoperability, and reusability for research software principles and compares reported use cases to framework functionalities to identify gaps in usability and scalability. The findings reveal that while most frameworks perform well in findability and reusability, they exhibit limited interoperability both among themselves and with specific software libraries. Although often developed for particular use cases, the technical foundations of these frameworks suggest potential for broader applicability. However, the scarce integration of privacy-preserving techniques and a predominant reliance on horizontal architectures may constrain their scalability in more complex federated learning scenarios. Ultimately, this analysis highlights the necessity for federated learning frameworks to evolve toward greater interoperability, flexibility, and privacy-awareness.

## Graphical Abstract

**Graphical Abstract:**
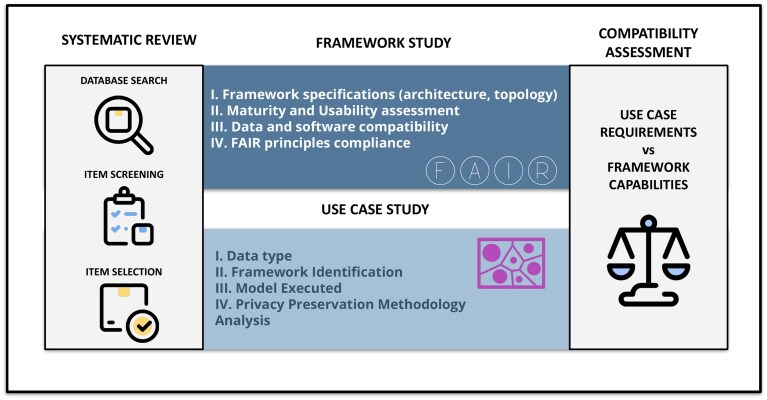

## Introduction

The increasing digitization of biomedical processes and the adoption of advanced technologies such as cloud computing in fields like precision medicine and biomedical informatics [1, 2] has led to unprecedented availability of enormous amounts of research data [3]. Given the sensitive nature of this data, using it for research purposes poses the challenge of ensuring its secure access and processing in an ethically sound, legally compliant, and technically efficient manner.

The broad adoption of artificial intelligence (AI) technologies, which refer to systems that learn patterns from data and make predictions or decisions, implies processing enormous amounts of data automatically without direct programming. In biomedicine, this technology is mainly used to analyze clinical, genomic, and medical image data, facilitating the early detection of diseases, the prediction of therapeutic outcomes, and the personalization of healthcare. As the amount of data generated by modern digital systems increases [3], traditional analysis techniques often need to be revisited to handle their heterogeneity, generation pace, and volume. Additionally, access to data, or even data transmission, is often limited by legal, ethical, privacy, and security concerns [4, 5], making it difficult to train AI models in a traditional centralized scenario. In these situations, Federated learning (FL) represents a solution to avoid compromising the privacy and security of data owners while accurate and robust models can still be obtained.

The concept of FL was first proposed by Google in 2016 [3, 6], and it can be defined as a paradigm of decentralized AI model training on distributed devices, eliminating the need to exchange raw data and, therefore, safeguarding the confidentiality and security of local information [1, 3, 7–10]. This approximation makes it possible to work with large amounts of data, using multiple data sources, taking advantage of the diversity of datasets present on each node [7], and avoiding all problems related to data transfer between nodes. Although an initial definition of FL was addressing the collection of data points from a great number of devices in real time, i.e. the “cross-devices” paradigm, the use of FL in Biomedicine is primarily “cross-silo” (organization level). Both approaches imply different challenges, but the second is more compatible with the data privacy and legal requirements currently accepted in biomedical settings [22].

Despite the significant potential of FL, several concerns and obstacles are accentuated in the context of biomedicine, both from a technical perspective and from aspects related to data governance and interoperability. In healthcare settings where sensitive biomedical information is generated and stored, data is often isolated because of legal protection purposes, often requiring specific processing to enable the secondary use of such data for research purposes [11]. Preparing biomedical data for research requires addressing both privacy and interoperability challenges. Anonymization is a critical step to prevent patient re-identification. Effective data utility depends on resolving syntactic and semantic interoperability barriers. Standardized data models like OHDSI OMOP and HL7 FHIR—paired with ontologies and controlled vocabularies—provide structured frameworks to harmonize disparate datasets [12]. These tools facilitate cross-institutional data alignment by defining common schemas and terminologies, which are essential for FL in healthcare settings where data remains decentralized [12, 13].

Data governance requires clear legal frameworks for the secondary use of data originally collected for primary purposes, such as patient care. Clarifying permissible research applications is critical, as interpretations of legal bases—including their scope and applicability—often differ between institutions and even across departments within the same organization. Thus, in order to limit these adverse effects, there has been an effort towards their regulation in the European context, also motivated by the need to establish standards and adequate governance models for data management. This is reflected in the enactment of the European Health Data Space (EHDS) [14] in 2025, which is composed of a set of rules and practices aimed at standardization for the exchange and access of health data at the EU level, and the AI Act [15, 16] also approved in 2024, aiming to balance research and industrial growth with trust and fundamental human rights, further supported by ethical guidelines outlined by the High-Level Expert Group on Artificial Intelligence (AI HLEG). Both initiatives are also affected by the European General Data Protection Regulation (GDPR) [17, 18], which aims to guarantee the security and privacy of the information gathered and exchanged on a more general level. All these regulations, including data-sharing policies derived from them, need to make use of federated infrastructures for managing and analysing the enormous amount of available data within these settings.

Additionally, for data providers to be effectively integrated into a federated network, they must have an adequate computational infrastructure of their own. Architectures of these data sites are usually heterogeneous, implying diversity in terms of hardware configurations, operating systems, and software, which adds an additional layer of complexity to the technical integration and interoperability of these infrastructures and inherently limits what models can be run on them.

Previous works on FL [4, 7, 8] have focused on particular topics rather than comprehensively addressing the interconnection of all the elements that make up a complete FL environment within a biomedical context. Therefore, this review focuses on FL models and infrastructures, aiming to illustrate the current state-of-the-art and functionalities of FL infrastructures, how they are represented in contemporary FL biomedical model settings, and how well they meet their needs.

In this review, we focus on FL frameworks, infrastructures, and their potential for effective implementation in real-world biomedical contexts. Specifically, we not only aim to assess the technical characteristics of existing FL infrastructures but also how these systems are, or could be, utilized to address practical biomedical challenges. To this end, we seek to answer three main research questions:

1. What are the current state and functionalities of FL infrastructures used in Biomedicine?
2. What are the types of use cases in which FL is being utilized in Biomedicine?
3. To what extent are privacy-preserving techniques used in current FL infrastructures and their applications?
4. To what extent do current FL infrastructures fulfil the requirements of these case studies?

## Material and methods

**Figure 1.**
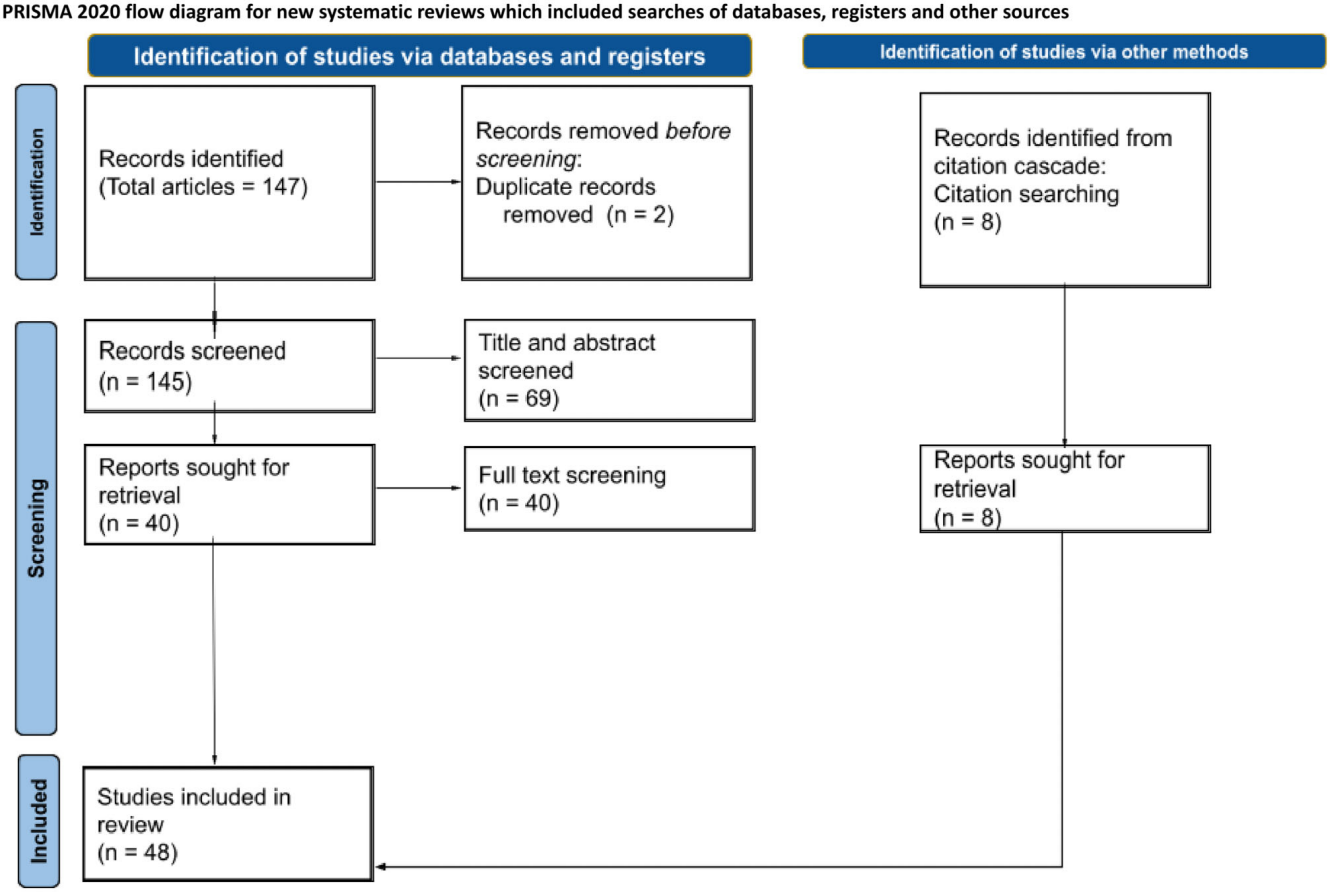
Overview of the manuscript inclusion process. This flow diagram is prepared following the “PRISMA 2020 flow diagram for new systematic reviews which included searches of databases, registers and other sources” template available at PRISMA Executive. (2024–2025). PRISMA 2020 flow diagram. PRISMA. https://www.prisma-statement.org/prisma-2020-flow-diagram

To collect the appropriate information, we have followed a systematic review approach [19] using the PRISMA guidelines [20]. Fig. 1 depicts the overview of the selection process of available works included in this review.

### Selection criteria

We defined four general categories as the main selection criteria to decide if a published manuscript could be of interest for this systematic review (see Table 1). This classification allowed us to refine the search keywords used (see next section “Search Strategy”).

**Table 1.** The four defined categories to decide if a manuscript could be of interest in this systematic review

Categories	
Overview	General articles on federated learning or reviews related to the topic.
Infrastructures	Descriptions of an original platform designed to perform FL analysis.
Case Study	Use cases where an FL analysis has been developed in the context of biomedical sciences.
Privacy Preservation	Manuscripts that tackle in depth the privacy preservation aspects related to an FL architecture or model.

### Search strategy

As previously stated, this systematic review was conducted in accordance with the PRISMA guidelines. To gather relevant information and to center the scope into the field of biomedical applications, we focused on two major databases widely recognized in the field of bioinformatics: PubMed (https://pubmed.ncbi.nlm.nih.gov/) and Web of Science. (https://webofscience.com). We have included publications from 2016 (when the FL concept was first introduced) until 2024.

The primary aim of this review is to assess the current state of infrastructures supporting the study of FL and its relevance to current research, while focusing on the four previously established categories, we selected the specific keywords “Federated Learning”, “FL for biomedical applications”, “FL infrastructure,” “Privacy preservation in FL,” “FL in biomedicine,” and “Differential privacy.” These keywords were combined using Boolean operators and were set to be found in journals belonging to the fields of “Health Informatics,” “Genetics,” “Medicine,” and “Biomedicine.” Additionally, only articles classified as “journal articles” and “reviews” were set for the search. This selection of keywords was intentionally kept narrow to limit the results to fields adjacent to biomedicine while maintaining a focus on applications of FL.

In addition to the established categorization criteria, several exclusion guidelines were also applied:

- Studies based on the principles of “distributed analyses” or “classical machine learning” instead of “federated learning”.
- Studies performed outside of the field of biomedicine (In this instance, biomedicine is used as an umbrella term that includes, among others, medicine, medical analysis, genetics, etc).
- Articles solely focused on “privacy preservation in AI” from a non-technical perspective.
- Conference abstracts and short papers.

### Data extraction

To determine what information to extract from each manuscript, the following specific focus areas were defined for each of the four main categories included in the review:

- **Overview:** In this category, no specific parameter was extracted; instead, a general approach was adopted in order to identify the core variables that define FL.
- **Infrastructures:** From the studies in this category, the following parameters were extracted: “Architecture”, “Topology”, “Number of citations”, “Maturity”, “Usability”, “Supported Libraries”, and “Compatible Data Types”. A more detailed explanation of the definition of these parameters can be found in the section Federated Learning Infrastructures.
- **Case Study:** For these articles, the extracted parameters were “Data type”, “Utilised framework”, and “Performed model”.
- **Privacy Preservation:** These methodologies are not the primary focus of this review. However, identifying and analyzing studies that address this aspect provides us with a more comprehensive understanding of the current state-of-the-art methodologies applicable to FL. This group of articles was fundamentally used to complement the others, helping to clarify what to focus on when either frameworks or use cases mentioned privacy preservation techniques.

## Results and discussion

**Figure 2.**
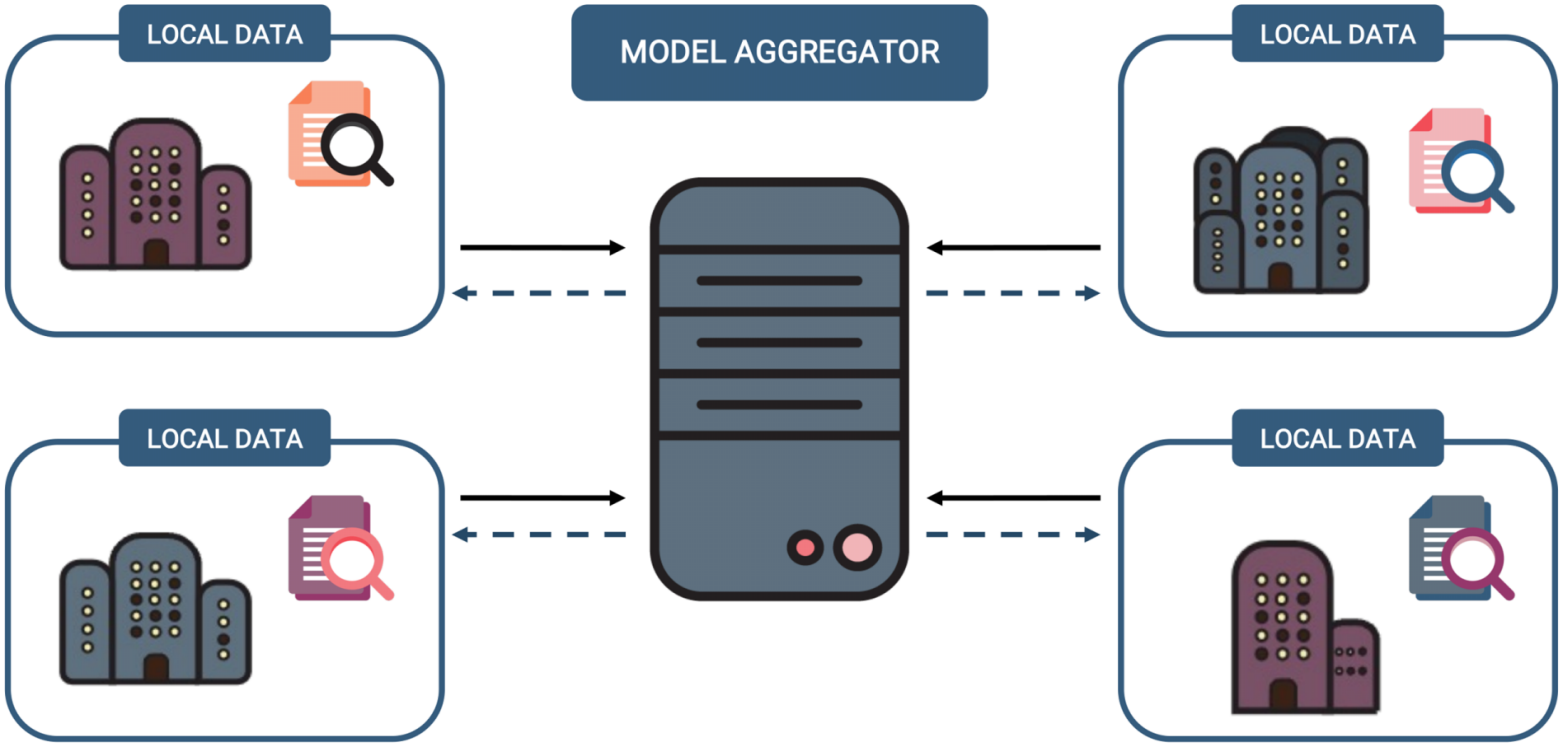
Representation of a traditional federated learning network, which is composed of a central model aggregator and several external provider nodes where local data are stored. In contrast to federated analysis, there are a number of communications between the model aggregator and the different nodes while generating the final AI model. Depending on how the infrastructure is built, the central aggregator can be located in an independent node or be hosted by one of the local nodes.

FL is defined as a decentralized [9] approach that enables the training of Artificial Intelligence (AI) models using data distributed across different sites without hoarding all the data in a single place (see Fig. 2). This process is not to be mistaken with the concept of federated analysis (FA), which only refers to the aggregation of the results [21] of an analysis performed in a distributed infrastructure, regardless of the analysis performed to obtain them.

In the context of biomedical research, this implies that instead of pooling sensitive data such as electronic health records, imaging data, or genomic information in a single computation node, FL allows each site that participates in the analysis (e.g. hospitals, laboratories, or research centers) to train a local copy of the model on its own data. In the most traditional network topology (Fig. 2), the resulting model parameters or weight updates are shared with a coordinating node, which aggregates them to create a global model, but no data is transmitted outside of the participating nodes. This enables institutions to collaboratively train models, ensuring compliance with privacy regulations. By leveraging diverse datasets from multiple sites, the output is more robust and generalizable [22, 23] models that also reduce the logistical challenges of centralizing biomedical data.

It is important to note that FL can be broadly classified into cross-device FL and cross-silo FL [24]. Cross-device FL involves a large number of clients, such as mobile or IoT devices, while cross-silo FL engages a number of institution-level participants. Given that biomedical data is typically stored within institutions and cannot be freely shared due to privacy and regulatory constraints, this work focuses on the cross-silo paradigm as the most suitable setting for collaborative model training in this context.

### Federated learning infrastructures

This first part of the review directs its focus towards a fundamental question of this study: **What is the current state and functionalities of FL infrastructures in Biomedicine?** (Question 1)

To address this question, it is important to first define fundamental concepts like what FL infrastructures, or frameworks, are. They refer to the collection of technical components that provide a structured, end-to-end environment for designing, deploying, and managing federated learning experiments. This includes all core services that provide the underlying support for software systems, enabling client-server coordination, secure communication, etc. For this reason, articles focused on the use of software libraries [25] have not been included in this analysis.

This section reviews a compilation of all the studies classified as part of the “Infrastructure” category, which is presented in Table 2, along with the data extracted from them. This compilation includes the characteristics of “Architecture” and “Topology,” which reference the data and physical distribution of a framework; “Maturity” as an inferred parameter based on Technology Readiness Levels (TRL) [26], “Usability,” which refers to an estimation of framework reproducibility level; “Supported Libraries” and “Generality,” which reference the kinds of data and libraries a specific framework can support.

**Table 2.** Analysis of the identified FL infrastructures

Framework	Year of publication	Number of citations	Reportedarchitecture	Supportedtopology	Maturity	Usability	Supported libraries	Compatible data types
SUBSTRA [30]	2019	57	Vertical	Decentralized	6	4	Agnostic to the training library	Images, Categorical data
MIP [31]	2020	14	Horizontal	Centralized	6	5	*No specific libraries mentioned*	Images
VANTAGE6 [32]	2020	85	Horizontal/Vertical	Centralized	8–9	4	*No specific libraries mentioned, but supports Python packages*	Images, Numerical data, Continuous data, Categorical data
FedN [33]	2021	65	Horizontal/Vertical	Hybrid	6	4	Keras	Images
OpenFL [34]	2021	148	Horizontal/Vertical	Centralized	8–9	4	TensorFlow, Keras, PyTorch	Images, Continuous data, Categorical data
NVIDIA-FLARE [35]	2021	774	Hybrid	Centralized	8–9	4	Agnostic to the training library	Images, Numerical data, Continuous data, Categorical data
APPFL [36]	2022	45	Horizontal	Centralized	6	4	*No specific libraries mentioned*	Images
Flower [37]	2022	1 363	Horizontal	Centralized	8–9	4	Pytorch, Tensorflow, Keras, and more	Images, Numerical data, Continuous data, Categorical data
Fed-Biomed [38]	2023	24	Horizontal	Centralized	7–8	4	PyTorch, Scikit-Learn, MONAI, and other Python libraries	Images, Numerical data, Continuous data, Categorical data
FedDNA [39]	2023	9	Horizontal	Centralized	3	1	*No specific libraries mentioned*	Images, Categorical data, Continuous data
FedMedChain [40]	2023	113	Horizontal	Decentralized	3	1	*No specific libraries mentioned*	Images, Continuous data, Categorical data
PerHeFed [41]	2023	4	Horizontal	Centralized	4	4	*No specific libraries mentioned*	Images
PrivaTree [42]	2024	12	Horizontal	Centralized	7	4	*No specific libraries mentioned, but Python*	Categorical data, Continuous data
FedAWA[43]	2024	16	Horizontal	Centralized	3	1	*No specific libraries mentioned, but supports Python packages*	Images
FKD-Med[44]	2024	41	Horizontal	Centralized	4–5	4	PyTorch	Images
Fed-CRFD[45]	2024	33	Vertical	Decentralized	4–5	3	Pytorch	Images
Deep-CFL[58]	2024	7	Horizontal	Centralized	3	1	*No specific libraries mentioned*	Categorical data, Continuous data

This table showcases the 17 frameworks that have been identified through this study. The **supported libraries** column indicates only the libraries specifically mentioned in the reference paper for each framework. The **compatible data types** showcased in this table are the following: Categorical data: variables that represent distinct groups or categories (e.g. blood type, gender, etc). Continuous data: numerical variables that can take on any value within a range, such as height or weight. Numerical data: values expressed as numbers that can be either discrete or continuous. E.g. Radiomics: visual information captured in the form of numerical data.

The application of FL models is closely related to the nature of distributed data that, in turn, forces the architecture of the FL network process. Different architectures can be broadly categorized into horizontal federated learning (HFL) and vertical federated learning (VFL), depending on how the data is distributed across participants. In HFL, multiple parties share the same feature space but differ in their data samples [8, 9, 27]. VFL, on the other hand, tackles a scenario where parties share the same sample space but with non-overlapping feature sets [8]. We can also differentiate hybrid federated learning architectures, which are an amalgamation of HFL and VFL.

Aside from architecture, FL infrastructures can be categorized regarding the physical arrangement of the resources that comprise their network structure [28]. Centralized topologies involve a central point of control, while decentralized topologies distribute control among multiple nodes, a category that would encompass those that present hierarchical control points. There are also peer-to-peer (P2P) and hybrid topologies, which offer an alternative to traditional server-centric frameworks [29]. P2P FL enables direct collaboration between devices without any kind of intermediaries, while hybrid topologies blend centralized and decentralized approaches together.

Aside from their architecture and topology, the values presented in Table 2 correspond to the following specific criteria:

- **Number of citations:** Number of times the specific paper we obtained the information from has been cited (until September, 2025).
- **Maturity**: assessed on a scale from 1 to 9, indicating the TRL level [26] of each infrastructure and reflecting the degree of technological development and stability, from conceptual design to validation in operational environments.
- **Usability**: measured on a scale of 1 to 5, evaluating how easily the infrastructure environment can be reproduced, with higher scores denoting greater ease of implementation and adoption by end users. For further explanation on how this value has been assigned, see Supplementary Material. Usability Scores
- **Library support**: refers to whether the infrastructure design is reported in its related paper to work with specific libraries such as PyTorch, Keras, and others.
- **Compatible data types and formats**: indicates the kinds of information supported by each framework, including categorical and continuous data, images, and other formats.

The results presented in Table 2 reveal that there is uniformity in the predominant architecture and topology of most of the analyzed frameworks. Specifically, 70.5% of the studied frameworks present a horizontal architecture, and 76.5% exhibit a centralized topology. This observed pattern makes sense because they simplify coordination and communication between a central server and its associated clients, making it easier to both manage data aggregation and perform model updates within the network.

A critical challenge in federated learning frameworks is achieving scalability while managing computational overhead. As the number of participating devices increases, maintaining synchronization, handling network latency, and preventing stragglers—devices that fall behind in updates—becomes increasingly difficult. Additionally, the computational requirements for training models across resource-constrained devices can significantly hinder performance. Many devices in federated settings may have limited processing power, memory, or battery life, which can lead to incomplete or inefficient training processes. Another major trade-off lies between privacy-preserving methods and model performance. Techniques such as differential privacy and secure multi-party computation (SMPC) help protect user data but often degrade the accuracy and efficiency of the global model due to added noise or higher computational costs, as it happens in SMPC. Finding the right balance between robust privacy guarantees and maintaining competitive performance remains a complex issue that requires continuous research and optimization. A more thorough investigation into these challenges can offer valuable insights for designing more resilient and adaptive federated learning systems.

It is important to note that two of the frameworks analyzed (FedAWA and Deep-CFL) did not have any repository or code available at the time of the study; the other two (FedDNA and FedmedChain) had their code no longer available at their original software repositories, since their respective publication, and another one (Fed-CRFD) presented accessibility restrictions. This means we are not capable of validating their proposed implementation, and we cannot assess the reproducibility and replicability of these frameworks.

**Figure 3.**
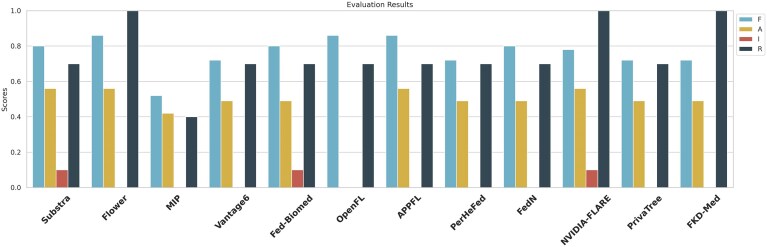
FAIRsoft scores for the 12 frameworks that could be evaluated. There are four scores—defined between 0 and 1—per framework representing each of the four FAIR principles. More information on how these values have been obtained can be found at Supplementary Material FAIRness evaluator.

To further understand the quality of these frameworks from the perspective of associated software metadata, we have assessed their FAIRness (see Fig. 3) using the methodology developed by Martin del Pico *et al.* [46]. FAIRsoft represents an automated measurement of a series of indicators derived from the findability, accessibility, interoperability, and reusability (FAIR) for Research Software principles [47]. These principles emphasise that, as it happens with research data, research software should be Findable, Accessible, Interoperable, and Reusable as necessary elements for sustainable high-quality software applications. Besides software availability and interoperability, proper license and existing documentation—both aspects under the Reusability principle—represent the minimum requirements that one would expect from functional frameworks that enable training and evaluating AI models in federated scenarios. As using a specific framework implies a significant investment of time and resources for researchers, choosing the right one is crucial for the success of any scientific project.

To facilitate the FAIRsoft indicators measurement, it is necessary to have access to a source code repository, e.g. GitHub, GitLab, BitBucket, etc., and software metadata available in at least one domain registry, e.g. bio.tools for Life Sciences [48]. Considering this requirement, information for only 12 out of the 17 studied frameworks was gathered and is further analyzed. Briefly, FAIRsoft indicators [46] combine automatically measured binary low-level indicators, e.g. existence of valid terms of use or a recognizable software license, that are combined into high-level indicators. Then, weighted high-level indicators are combined to provide an overall quantitative value for each of the four reference areas (FAIR). The resulting quantitative assessment offers insights into the degree of FAIR compliance, facilitating the identification of trends and areas for enhancement over time.

As previously found for general bioinformatics software [46], applying FAIRsoft metrics to federated learning frameworks reveals divergent performance across principles. Findability scores (0.7–0.9) and reusability (0.7–1.0, excluding MIP at 0.4) are strongest, reflecting frameworks’ clear licensing, documentation, and discoverability. MIP’s lower reusability is expected, as it was developed to fulfill a specific use case that limits its adaptability. Accessibility scores (0.4–0.6) indicate basic compliance, e.g. frameworks are downloadable and buildable, but lack many advanced features like test datasets, multi-platform support, or integration with diverse e-infrastructures.

Interoperability scores are notably weak, with only Substra and Fed-Biomed documenting software dependencies. This underscores a systemic gap in horizontal interoperability, the ability to interact with peer frameworks, which aligns with broader FL challenges in scaling across heterogeneous systems. The findings highlight an urgent need for standardized interfaces and dependency management to enable cross-framework collaboration in federated ecosystems.

Evaluating the FAIRness of software infrastructures offers a novel approach to assessing software quality. Software metadata plays a crucial role in ensuring the longevity of any system, and FAIRsoft indicators provide a valuable mechanism to measure metadata quality, serving as an effective proxy for overall software robustness. Long-term maintenance, adaptation, and extension of software depend heavily on such quality considerations. Given the current lack of interoperability among federated learning frameworks, a thorough evaluation of these factors is essential before selecting a framework, as adoption requires substantial time and resource investment across the entire federation supporting such analyses.

### Contemporary FL case studies

The second question of this review revolved around **What are the type of use cases in which Federated Learning is utilized in Biomedicine?** (Question 2)

The following table (Table 2) shows the compilation of articles belonging to the category of use cases, where the identified parameters were the type of data used in each study, the mention or explanation of the framework used, as well as the type of model and the privacy preservation methodology implemented in each study, where we are differentiating differential privacy (DP) [4, 18], secure multiparty computation (SMPC) [27, 49], symmetric encryption [50], and homomorphic encryption [51, 52].

Table 3 shows that 12 out of the 22 reviewed studies (54.5%) provide a solution for image analysis. The second most popular category relates to Categorical and Continuous data, which are typically present in Electronic Health Records (EHRs). This type of data is used as input in eight out of the 22 studies (36.4%). These types of datasets are very common when conducting clinical studies, so it is not surprising to see that the vast majority of use cases are carried out using one of them. These datasets show a correlation with the models used in the studies. Around 16 of the 22 studies (72.7%) identify neural networks as their underlying model, which is consistent, considering that this is a widely used approach in medical imaging research when assembling AI models.

**Table 3.** Analysis of the identified case studies

Case studies	Data type	Framework	Model
Abbas, *et al.* 2023 [53]	Images	Not Specified	NN
Chen, *et al.* 2021 [27]	Categorical data	Not Specified	NN
Dayan, *et al.* 2021 [35]	Categorical data, Continuous data, Images	NVIDIA-FLARE	DNN
El Zein *et al.* 2024 [42]	Categorical data, Continuous data	PrivaTree	Decision Tree
Hauschild, *et al.* 2022 [54]	Numerical data	Not Specified	Federated Random Forest
Jimenez-Sanchez, *et al.* 2023 [55]	Images	Not Specified	CNN
Lee, *et al.* 2020 [56]	Categorical data, Continuous data	Not Specified	ANN
Lin, *et al.* 2023 [57]	Images	Not Specified	DNN
Ma, *et al.* 2023 [41]	Images	PerHeFed	CNN
Nguyen *et al.* 2024 [58]	Categorical data, Continuous data	Deep-CFL	RNN
Rajendran, *et al.* 2021 [59]	Categorical data, Continuous data	Not Specified	ANN, LR
Redolfi, *et al.* 2020 [31]	Images	MIP	MANOVA
Sadilek, *et al.* 2021 [60]	Categorical data, Continuous data	Not Specified	NN, DNN, LR
Samuel, *et al.* 2023 [40]	Categorical data, Continuous data	FedMedChain	DNN
Sav, *et al.* 2022 [51]	Images	Not Specified	CNN
Sheller, *et al.* 2020 [61]	Images	Not Specified	CNN
Sun *et al.* 2024 [44]	Images	FKD-Med	CNN
Wang S, *et al.* 2023 [39]	Numerical data	FedDNA	NN
Wang Z, *et al.* 2022 [62]	Images	Not Specified	NN
Wang, *et al.* 2022 [63]	Categorical data, Continuous data	Not Specified	LR
Yan *et al.* 2024 [45]	Images	Fed-CRFD	CNN
Yue *et al.* 2024 [43]	Images	FedAWA	DNN

This table showcases the 22 papers classified as “case studies.” For each one of them, the data type, model, and framework utilized to carry out the analyses are highlighted. The value **Not Specified** indicates that a specific parameter is not identifiable. In the model column, the following acronyms are being used: LR: Linear Regression. NN: Neural Network. CNN: Convolutional Neural Network. DNN: Deep Neural Network. ANN: Artificial Neural Network. RNN: Recurrent Neural Network

Out of the 22 studies reviewed, only six (27.2%) explicitly reported using one of the frameworks listed in Table 2. In all but one of these cases (Dylan *et al.*, which utilized NVIDIA-FLARE), both the use case and the corresponding framework were described within the same publication. The remaining 16 studies did not specify the framework employed. However, the absence of this information does not necessarily indicate that no framework was used. Rather, it suggests that either the infrastructure details were not disclosed within the case study descriptions, or that a custom, ad-hoc infrastructure was used specifically for the study and never transformed into a stable product.

Additionally, the information gathered through this table allows us to tackle the third question of this review: **To what extent are privacy-preserving techniques used in current FL infrastructures and their applications?** (Question 3).

**Figure 4.**
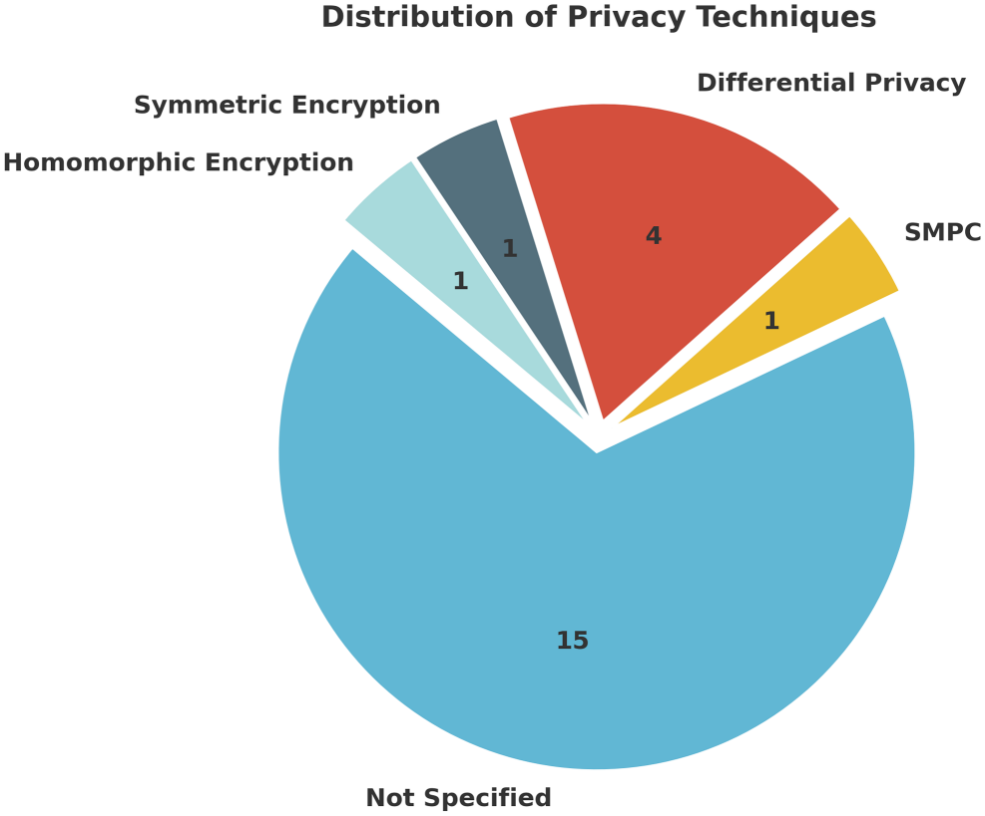
Number of privacy preservation techniques implemented in various case studies, including homomorphic encryption, symmetric encryption, differential privacy, and SMPC. It also showcases how many of their use cases are not implementing, or at least are not reporting, any privacy preservation technique.

It is observed that only seven of the 22 studies reported implementing privacy preservation techniques (see Fig. 4). Of these, seven (31.8%) provided detailed descriptions of the security protocols utilized. Among them, three employed differential privacy (DP), two used encryption-based protocols, and one implemented secure multi-party computation (SMPC).

The low proportion of studies addressing the security and privacy preservation aspects could be attributed to the focus of the articles on the results produced by the implemented models rather than a precise description of the model environment. We also found that the only frameworks that elaborate about privacy preservation are NVIDIA-FLARE and FedMedChain. It would be relevant to investigate the implications and results that would be derived from the implementation of these privacy preservation techniques in these specific case studies that make use of the aforementioned frameworks. Additionally, there is a consensus in the literature that more research is needed on privacy-preserving FL architectures that take into account the different trade-off requirements in terms of convergence performance and privacy levels [3, 9, 60, 64].

### Inferred compatibility of contemporary FL infrastructures and case studies

The final question of the review was to determine **to what extent current FL infrastructures fulfill the requirements of these case studies?** (Question 4)

To assess the feasibility of utilizing the frameworks presented in Table 2 for implementing the specific use cases outlined in Table 3, and to evaluate how effectively current FL infrastructures address the requirements of these case studies, a correlation analysis between the two previously introduced tables was conducted, considering the following factors:

- Compatibility with the type of executed model: It was evaluated whether the frameworks mentioned in Table 2 are theoretically compatible with the type of executed model required by each use case in Table 3, either because the framework explicitly supports the type of model used in the case studies, or it is compatible with libraries from which the same type of model can be executed.
- Data type admissibility: It was examined whether the frameworks can handle the data types used in each specific use case.

In cases where a specific library is not mentioned in the use case study (e.g. a study showcasing DNNs), we assumed that the analysis could be replicated using frameworks that include libraries supporting this type of model, such as PyTorch or Keras. If a framework does not specify which libraries it uses but is compatible with the data type of the use case, we assume it possesses a general degree of compatibility and that the use case could be developed within the framework’s environment, though practical implementation may depend on the particular requirements of each case.

In addition, and for the purpose of a more seamless analysis, unless stated otherwise in the corresponding use case paper, it is assumed that the data distribution in the referenced studies follows a traditional FL scenario, i.e. fitting a horizontal architecture and centralized topology. In cases where we are faced with hybrid architectures that would otherwise fill the case study requirements, we would have to evaluate each specific scenario to see how the framework would need to adapt, granting them a general degree of compatibility.

It is important to note that the analysis performed is theoretical and relies on the evidence presented across the reviewed literature rather than on empirical experimentation. For that reason, frameworks whose source code is currently unavailable are still included in this analysis. Nevertheless, this comparison remains significant because it enables us to critically assess how the reported infrastructures align with the needs of biomedical use cases, to identify potential gaps, and to highlight directions where practical implementation could be improved.

These observations suggest both thematic and methodological convergence among most of the infrastructures and case studies examined, which is reinforced by the compatibility analysis between frameworks and case studies performed in Table 4. According to the data collected in Table 4, it is observed that, of the 17 infrastructures evaluated in Table 2, a total of six of them exhibit attributes that potentially align them with more than 50% of the use cases detailed in Table 3, based on their compatible model libraries and evaluated data inputs.

**Table 4.** Case studies and FL infrastructures comparative

	Frameworks																
Case Studies	SUBSTRA	FedDNA	MIP	VANTAGE6	PerHeFed	FedMed Chain	Fed-Biomed	OpenFL	APPFL	FedN	Flower	NVIDIA-FLARE	PrivaTree	FedAWA	FKD-Med	Fed-CRFD	Deep-CFL
Abbas, *et al.* 2023		Gen	Gen	Gen	Gen		Comp	Comp	Gen	Comp	Comp	Comp		Gen	Comp		
Chen, *et al.* 2021		Gen		Gen			Comp	Comp			Comp	Comp	Gen				Gen
Dayan, *et al.* 2021		Gen	Gen	Gen	Gen		Comp	Comp	Gen	Comp	Comp	Feat		Gen	Comp		
El Zein *et al.* 2024		Gen		Gen			Comp	Comp			Comp	Comp	Feat				Gen
Hauschild, *et al.* 2022				Gen			Comp				Gen	Gen	Gen				
Jimenez-Sanchez, *et al.* 2023		Gen	Gen	Gen	Gen		Comp	Comp	Gen	Comp	Comp	Comp		Gen	Comp		
Lee, *et al.* 2020		Gen		Gen			Comp	Comp			Comp	Comp	Gen				Gen
Lin, *et al.* 2023		Gen	Gen	Gen	Gen		Comp	Comp	Gen	Comp	Comp	Comp		Gen	Comp		
Ma, *et al.* 2023		Gen	Gen	Gen	Gen		Comp	Comp	Gen	Comp	Comp	Comp		Gen	Comp		
Nguyen *et al.* 2024		Gen		Gen			Comp	Comp			Comp	Comp	Gen				Feat
Rajendran, *et al.* 2021		Gen		Gen			Comp	Comp			Comp	Comp	Gen				Gen
Redolfi, *et al.* 2020		Gen	Feat	Gen	Gen		Comp	Comp	Gen	Comp	Comp	Comp		Gen	Comp		
Sadilek, *et al.* 2021		Gen		Gen			Comp	Comp			Comp	Comp	Gen				Gen
Samuel, *et al.* 2023						Feat											
Sav, *et al.* 2022		Gen	Gen	Gen	Gen		Comp	Comp	Gen	Comp	Comp	Comp		Gen	Comp		
Sheller, *et al.* 2020		Gen	Gen	Gen	Gen		Comp	Comp	Gen	Comp	Comp	Comp		Gen	Comp		
Sun *et al.* 2024		Gen	Gen	Gen	Gen		Comp	Comp	Gen	Comp	Comp	Comp		Gen	Feat		
Wang S, *et al.* 2023		Feat	Gen	Gen	Gen		Comp	Comp	Gen	Comp	Comp	Comp		Gen	Comp		
Wang Z, *et al.* 2022		Gen	Gen	Gen	Gen		Comp	Comp	Gen	Comp	Comp	Comp		Gen	Comp		
Wang, *et al.* 2022		Gen		Gen			Comp	Comp			Comp	Comp	Gen				Gen
Yan *et al.* 2024	Gen															Feat	
Yue *et al.* 2024		Gen	Gen	Gen	Gen		Comp	Comp	Gen	Comp	Comp	Comp		Feat	Comp		

This table showcases the 17 papers classified as “case studies” from Table 2 and links them to each one of the chosen infrastructures showcased in Table 1. The value *Comp* indicates theoretical compatibility (e.g. common libraries) between them, the value *Gen* indicates the scenario of a general degree of compatibility (i.e. data compatibility), and the value *Feat* indicates that the infrastructure is showcased in the study, it is linked to.

There are three frameworks that present little to no compatibility: Substra, Fed-CRFD, and FedMedChain. With the gathered information, Substra only has one compatible use case listed in the reference table, as it implements a vertical architecture combined with a decentralized topology. Similarly, Fed-CRFD is based on a vertical architecture, and FedMedChain presents a purely decentralized topology, which limits their direct compatibility with the reviewed use cases. This does not imply that the presented use cases could not be implemented using these three frameworks; rather, it indicates that further investigation is required to determine whether the corresponding models could be effectively adapted to operate under those alternative frameworks. An example of this is the work by Heyndrickx *et al.* [65] in 2024, in which they have used Substra for an HFL-based use case. Although Substra was originally not described for horizontal FL studies [30], his case underscores how software frameworks can evolve over time, expanding their capabilities and supporting a broader range of applications as their ecosystems mature.

The overall showcased trend would indicate that the present development of FL frameworks is largely use case driven, providing ad-hoc solutions opposite to generating a general-purpose infrastructure. Furthermore, as the biomedical data types for which FL models are being developed are relatively small (primarily medical images and data associated with EHRs), most solutions would be largely compatible, as the models would need to use the same set of software libraries, mainly written in Python. As noted earlier, this compatibility analysis is theoretical, and confirming its validity would involve replicating the original experiments. This would require access to both the original underlying code and data, resources that are not currently available.

## Conclusions

The rise of artificial intelligence across scientific disciplines is accelerating the development of technological solutions to use massive amounts of data across geographically distributed sites. Among those solutions, federated learning offers the possibility to train and evaluate AI models across sites and organizations. Considering the sensitive nature of data used in biomedical research, there are strict regulations in terms of what data can be accessed and used for, not just from a legal point of view but also from ethical considerations. Therefore, federated learning represents a feasible option to process enormous amounts of data without mobilizing datasets from their sources, given that access is granted. However, as with any emerging technological development, the field is under constant change, which implies that new solutions are made publicly available every few months, preventing the consolidation of existing ones. This review puts the focus on existing infrastructures, here also named frameworks, and examines them in terms of quality, as a proxy of long-term sustainability, and use as a function of different use cases. As biomedical data is, in most cases, managed at the institutional level, we have put the focus on the challenges and alternatives to deal with FL across institutions (cross-silo paradigm), leaving aside the challenges associated with cross-devices. The emergence of Patient-Reported Outcome Measures (PROMs) might lead to FL scenarios where the challenge is to manage the computational requirements to synchronize millions of devices to train, validate, and use AI models. Following common practices in biomedicine, we have only used publications classified as “journal articles” and “reviews.” This selection was intentionally kept narrow to limit the results to fields adjacent to biomedicine while maintaining a focus on applications of FL. Therefore, we have explicitly excluded potentially relevant publications from conference abstracts and short papers, which is the common practice to fields like Computer Sciences to communicate new findings and developments.

### General applicability of FL infrastructures

An important finding is that many of the analyzed frameworks were created after a specific case study, which guarantees their usability in at least one experiment but also limits their flexibility and modularity. The more flexible a framework is, the more experiments can be conducted using such a framework, which should contribute towards its maintenance and, overall, long-term sustainability. Beyond the usual indicators like usability and maturity, we have applied here the FAIRsoft high- and low-level indicators associated with the FAIR for Research Software principles [40]. Understanding the FAIRness of research software represents a dimension of quality, which is intimately linked to its long-term sustainability. As in previous studies [39], frameworks scored reasonably well for findability and re-usability, moderately for accessibility, and poorly for interoperability. This implies that frameworks can be unequivocally found (findability) and have reasonable documentation and clarity in terms of licences (re-usability) and can be built/downloaded and deployed across different sites using at least one operating system (accessibility), but are not designed to interoperate between them (horizontal interoperability). In terms of vertical interoperability, some of them support the use of specific packages and libraries, e.g. Pytorch, Keras, etc.; although this is not broadly documented and available until a user decides to use them. As each site might have different technical requirements, including cybersecurity ones, choosing which framework is supported is critical. Indeed, in an ideal scenario, the focus should be put into increasing the interoperability across frameworks to favour their joint use to lower the adoption barrier by the data providers and processing computational facilities.

### Data Interoperability aspects

When considering interoperability aspects, there is the assumption that research data used for FL experiments is semantically interoperable across sites. However, this is only possible if specific actions are taken across participating sites to guarantee that level of interoperability. Specifically, it implies that agreements should be reached on which standard, e.g. OMOP or FHIR, should be used to model available data and in which format, e.g. XML, JSON, or SQL tables. Once this agreement is reached, each participating site should extract and harmonize available data before conducting quality checks as part of the pre-processing pipelines used to feed data to FL experiments.

### FL infrastructures in biomedical use cases

We confronted a number of use cases reported in the literature with the available frameworks to further understand their usability. We took the functionality of each framework and the experiments performed within the use cases, and evaluated whether it would have been possible to conduct such experiments with each of the available frameworks (Table 4). To our surprise, we discovered a significant theoretical gap between the capabilities offered by existing frameworks and the solutions actually implemented in the reported use cases. Despite the availability of ready-made tools designed to execute specific models and tasks, most implementation scenarios opted to develop custom solutions instead. This trend highlights a clear need for further research aimed at optimizing and standardizing these frameworks, ensuring they better meet the practical needs of the community. Another important aspect to consider is the scalability of the evaluated frameworks in real-life scenarios to fully understand the capabilities and potential limits of those infrastructures in terms of data management and model inferences. Such an evaluation would guide interested parties in using one or another framework, depending on the experiments to be conducted, given that some frameworks support similar libraries and have been used in similar use cases, e.g. medical imaging analysis.

### Identified gaps and outlook perspectives

Different gaps were identified when broadening the intersection of the experiments conducted within the use cases with the available frameworks. First, despite the potential configurations of some frameworks, for most of the experiments, data was horizontally available, e.g. same data types were available in all participating nodes, and orchestration was centrally done, e.g. a dedicated node was responsible for driving the interactions with the rest of the nodes. Therefore, it would be necessary to explore the opportunities and limitations of non-horizontal architectures, e.g. heterogeneous data types available across the different sites, and non-centralized topologies, e.g. use of peer-to-peer connections, robin-round assignments of the aggregation node. Second, limited attention has been devoted to privacy preservation techniques within use cases using federated learning. This finding suggests an underdeveloped area of research and highlights the need for future studies exploring the challenges and opportunities of applying such techniques, especially considering that data normally remains at each source without being mobilized.

As artificial intelligence keeps rising and diversifying, it is important to further investigate the intersection between data, models, and infrastructures for federated learning. Federated learning represents an incredible enabler for using highly regulated biomedical data for research purposes, which reduces the technological barriers for processing enormous amounts of data across different sites and organizations.

## Supplementary Material

lqag010_Supplemental_File

## Data Availability

The data underlying this article are available in the article and in its online supplementary material.

## Code availability

The code for the FAIR evaluation using FAIRSoft can be found in the following repository: https://doi.org/10.5281/zenodo.18183790.
