# Supplementary material for: Federated learning frameworks: quality and interoperability for biomedical research
Source: NAR Genom Bioinform. 2026 Feb 2;8(1):lqag010. doi: 10.1093/nargab/lqag010 (PMC12862364; doi:10.1093/nargab/lqag010)
Supplement: lqag010_Supplemental_File [file lqag010_supplemental_file.docx]

# **Supplementary Materials to Federated Learning Frameworks: Quality and Interoperability for Biomedical Research**

María Chavero-Diez^1,2^, Carles Hernandez-Ferrer^1^, Laia Codó^1^, Josep Ll. Gelpí^1,2,^, Salvador Capella-Gutiérrez^1,*^

*1. Barcelona Supercomputing Center, Plaça d’Eusebi Güell, 1-3, 08034 Barcelona, Spain. 2 Biochemistry and Molecular Biomedicine Department, University of Barcelona, , Av. Diagonal 643, 08028 Barcelona, Spain.*

**Usability Scores**

The following levels indicate the usability score given to each framework showcased on Table 2:

- Level 1: No documentation found
- Level 2: There is documentation but it's private/not everyone can access it
- Level 3: There is documentation publicly available, but not any tutorial or instruction to install or replicate the implementation.
- Level 4: There is documentation publicly available, and there is enough material to make the framework installable or replicable if you have technical knowledge, i.e: Github repository how-tos.
- Level 5: Anyone can download and set up their own environment without technical knowledge .

## **Framework Repositories**

In order to perform the FAIR analysis on the selected frameworks, we used the public code repositories available from each framework (see **Supplementary Table 1**)

***Supplementary table 1***. Links to each one of the available code repositories per infrastructure. Those that did not have them available are marked as X.

| **Framework** | **Github Repository** |
| --- | --- |
| APPFL | <https://github.com/APPFL/APPFL> |
| Fed-Biomed | <https://github.com/fedbiomed/fedbiomed> |
| FedDNA | Not available |
| FedMedChain | Not available |
| FedN | <https://github.com/scaleoutsystems/fedn> |
| Flower | <https://github.com/adap/flower> |
| MIP | <https://github.com/HBPMedical/mip-deployment> |
| OpenFL | <https://github.com/securefederatedai/openfl> |
| PerHeFed | <https://github.com/haroroda/PerHeFed> |
| SUBSTRA | <https://github.com/Substra/substrafl> |
| VANTAGE6 | <https://github.com/vantage6/vantage6> |
| NVIDIA-FLARE | <https://github.com/NVIDIA/NVFlare> |
| PrivaTree | <https://github.com/tudelft-cda-lab/PrivaTree> |
| FedAWA | Not available |
| FKD-Med | <https://github.com/SUN-1024/FKD-Med> |
| Fed-CRFD | <https://github.com/IAMJackYan/FedCRFD> |
| Deep-CFL | Not available |
